# Olfactory cues and the value of information: voles interpret cues based on recent predator encounters

**DOI:** 10.1007/s00265-018-2600-9

**Published:** 2018-11-26

**Authors:** Sonny S. Bleicher, Hannu Ylönen, Teemu Käpylä, Marko Haapakoski

**Affiliations:** 1 Environmental Science and Policy Department, George Mason Univesity , Fairfax, VA 22030 USA; 2 Konnevesi Research Station, University of Jyväskylä, P.O. Box 35, 40014 Jyväskylä, Finland

**Keywords:** Predator-prey interactions, Giving-up density, Perceived risk, Evolutionary game theory, Y-maze

## Abstract

**Abstract:**

Prey strategically respond to the risk of predation by varying their behavior while balancing the tradeoffs of food and safety. We present here an experiment that tests the way the same indirect cues of predation risk are interpreted by bank voles, *Myodes glareolus*, as the game changes through exposure to a caged weasel. Using optimal patch use, we asked wild-caught voles to rank the risk they perceived. We measured their response to olfactory cues in the form of weasel bedding, a sham control in the form of rabbit bedding, and an odor-free control. We repeated the interviews in a chronological order to test the change in response, i.e., the changes in the value of the information. We found that the voles did not differentiate strongly between treatments pre-exposure to the weasel. During the exposure, vole foraging activity was reduced in all treatments, but proportionally increased in the vicinity to the rabbit odor. Post-exposure, the voles focused their foraging in the control, while the value of exposure to the predator explained the majority of variation in response. Our data also suggested a sex bias in interpretation of the cues. Given how the foragers changed their interpretation of the same cues based on external information, we suggest that applying predator olfactory cues as a simulation of predation risk needs further testing. For instance, what are the possible effective compounds and how they change “fear” response over time. The major conclusion is that however effective olfactory cues may be, the presence of live predators overwhelmingly affects the information voles gained from these cues.

**Significance statement:**

In ecology, “fear” is the strategic response to cues of risk an animal senses in its environment. The cues suggesting the existence of a predator in the vicinity are weighed by an individual against the probability of encounter with the predator and the perceived lethality of an encounter with the predator. The best documented such response is variation in foraging tenacity as measured by a giving-up density. In this paper, we show that an olfactory predator cue and the smell of an interspecific competitor result in different responses based on experience with a live-caged predator. This work provides a cautionary example of the risk in making assumptions regarding olfactory cues devoid of environmental context.

**Electronic supplementary material:**

The online version of this article (10.1007/s00265-018-2600-9) contains supplementary material, which is available to authorized users.

## Introduction

Perhaps one of the greatest questions occupying behavioral ecologists, as well as some neurobiologists today, is how animals interpret information they gather in their environment (e.g., Dielenberg and McGregor 2001; Zimmer et al. 2006; Pakanen et al. 2014; Drakeley et al. 2015). In many instances, animals born in the lab, even first generation, exhibit weakened responses to predators which they would encounter on a day-to-day basis in nature (e.g., Burns et al. 2009; Feenders et al. 2011; Troxell-Smith et al. 2015). Similarly, but on a larger scale, ecologists have been puzzling over the inability of prey to recognize risk from unfamiliar, invasive, predators (Carthey and Banks 2014). From the opposite side, some wild species, such as Australian bush rats (*Rattus fuscipes*), have been shown to exhibit stress when exposed to urine and fur of red foxes (*Vulpes vulpes*) despite sharing no evolutionary history with any foxes (Banks 1998; Spencer et al. 2014). These examples have led to an increased debate on the value of information gained from cues of predation risk, especially in the absence of an actual predator.

To bring an example of this debate, we once more return to the prey naiveté literature and to the Australian researchers who pioneer this research. Banks and Dickman (2007) offer a framework to think about how prey interpret risk cues of novel predators as three levels of naiveté. They argue that multiple behavioral mechanisms are acting to prevent proper interpretation and responses to those novel risks. The first is the lack of a neurological pathway to recognize the cue as a predator cue altogether (e.g., Blumstein et al. 2000; Carthey et al. 2017). The second level is a mismatch between the interpretation of the cue and the behavioral response. The best example for this type of naiveté is New Zealand’s kakapo parrot (*Strigops habroptilus*) that recognizes cats (*Felis catus*) as predators but stares them down as opposed to fleeing (Karl and Best 1982). Last, they argue that simply being bad at using your tools against the novel predator is a form of naiveté, and in our opinion, that is still up for debate.

We began this introduction on the broad and applied implications of responding to predator cues because “fear” responses are some of the strongest traits inherited from ancestors. Anti-predator traits currently used by living beings were refined by millions of years of necessity to avoid predation (Vincent and Brown 2005). Otherwise, the current species would have experienced extinction along their line of decent (Darwin 1859). Based on this simple Darwinistic logic, we expect and assume that animals would recognize the odor of their evolutionarily known predators as a sign of risk. This logical assumption is still controversial.

Some studies, both in the laboratory and in the field verified the expected responses of prey to scent-mediated increase in predation risk from known predators (Ylönen and Ronkainen 1994; Koskela et al. 1997; Fuelling and Halle 2004; Ylönen et al. 2006). However, an equally large number of studies have found weak or no correlations between olfactory cues and anti-predator behaviors (Mappes et al. 1998; Trebatická et al. 2012). The ecological literature is filled with examples of predator odor impacting the behavior of prey in varying ways. For example, small mammal traps sprayed by odor, regardless if predator or conspecific, yield better trapping success than odorless traps (Apfelbach et al. 2005). Passerines will nest in boxes sprayed with weasel scent when competition for odor-free boxes gets high. However, in this case, the stress in the weasel scent–sprayed boxes resulted in increased fledging rate (Monkkonen et al. 2009). But perhaps one of the strongest examples of a mismatch is derived from native Australian marsupials which fail to appropriately respond to dingo scents, for example, the western gray kangaroos that instead of avoiding the predator odors are drawn to them (Parsons et al. 2005; Mella et al. 2014).

So why do we find variations in response to predator odor? A recent review by Parsons et al. (2018) formulated the pathways by which predator odors function and are recognized. They state that three complementary catalysts can generate a response to such cues: neurobiological, chemical, and contextual. They suggest that these interact to determine whether a scent is perceived as risky or attractive. Thus, we assume that the information gleaned from the olfactory cue can be interpreted in a different manner in different environmental or temporal context, in different habitats, and under different social regimes. We speculate that human researchers underestimate the cognitive and neural abilities of prey individuals to interpret the information in a cue. In colloquial and anthropomorphic terms, we can observe a number of examples: (1) Not “Danger: a predator is here!”, but “A predator was here a while ago and I can do whatever until it comes back”; (2) Not “I smell predator X therefore I must run or hide”, but “Predator X poses Y amount of risk to me. Therefore, I can take some risk to get food my competitors may avoid.”

The reason we can speculate that the responses are more complex lies in the basic difference between the neurological approach to predation risk (and post-traumatic stress) and that of behavioral ecology. Neurology approaches “fear” as the activation of a pathway between the hippocampus and the amygdala following an unforeseen exposure to a risk cue (Gross and Canteras 2012), i.e., as the response to an “Act of God”. However, as ecologists, we must argue here that an anti-predator behavior is more than an instinctive freezing or fleeing response. Instead, it is a strategic response based on the innate and acquired information an animal processes in the cerebral cortex, which in turn influences and regulates the production of stress hormones in the amygdala (Brown 2010).

In this paper, we set out to test how the interpretation of an olfactory cue may change based on the available information a prey individual has on the acute danger from predators in close vicinity. Using an interview chamber approach (cf. Bleicher 2014; Bleicher and Dickman 2016), we aim to “ask” the animals how they perceived the difference between olfactory cues of a predator, a cue of a competitor (herbivore) as a sham control, and a cue-less control. We request the forgiveness of some sensitive readers for the use of colloquiality, but we perceive this anthropomorphism (an interview) to be appropriate in this context. We apply this method (a type of bioassay) to determine how an individual animal perceives a “question” we pose. We then follow-up with repeated measures to address how the individual’s perception changes over time or based on the experience we subject it to. In this case, we interview individuals to assess how they interpret the information posed by an olfactory cue of a predator. We are interested in understanding the changes in an animal’s perceived risk on three scenarios: (1) after a few weeks in the lab and before exposure to a live predator, (2) while being exposed to a caged predator in an adjacent room, and (3) after that exposure. We decided to approach this example using a model system studied for decades, the bank vole (*Myodes glareolus*) and the least weasel (*Mustela nivalis nivalis*) as its predator (cf. Korpimäki et al. 1996; Sundell et al. 2008).

In this experiment, we expect that the voles would use resources optimally, balancing the tradeoffs of food and safety (cf. Brown 1988). We hypothesize that the odor of a predator would cause the voles to increase their vigilance and thus reduce their foraging in patches (cf. Brown 1999). We also expect the interpretation of the predator’s cue to change based on the exposure to the predator. From other empirical studies with small mammals, we can expect that exposure to a predator can have a lingering effect in the apprehension sensed by the prey (Dall et al. 2001). Last, we hypothesize that the energy needs, and the corresponding behavioral response, of the two sexes would not be the same. In the bank vole, the females are territorial and take the brunt of the energetic costs of reproduction while males move through the landscape in search of copulations (Horne and Ylönen 1996; Trebatická et al. 2012). In early spring, at the start of the breeding season, this sex bias in energetic tradeoffs should be at its peak. Therefore, we expect that the males would show greater apprehension around the predator cues as their transient nature exposes them to more predation risk than the females who are more sessile in their territories and resource focused at this time of year.

Given these expectations, we aim to answer four questions in this three-stage experiment:

1. Do voles differentiate between the odor of a predator and that of a competitor? If so, do they forage less in the presence of the odor of a predator than that of a competitor?
2. Does recent exposure to the predator invigorate a response to an olfactory cue of that predator?
3. Does information about an active predator nearby affect the activity patterns of the prey species in relation to both the predator and the competitor cues?
4. Does the information about a live predator linger post-exposure? If so, for how long?

## Methods

### Study species

The bank vole is one of the most common small rodents in northern temperate and boreal forests (Stenseth 1985). It is granivorous-omnivorous (Hansson 1979) and can live in a wide range of forest habitats. In central Finland, bank voles are known to breed between three and five times within the breeding season, May through September. Their average litter size is 5–6 pups. Bank voles are prey for a diverse predator assemblage which includes the least weasel and the stoat (*Mustela erminea*) (Ylönen 1989).

The least weasel is a specialist predator on rodents and the major cause of mortality in boreal voles, especially during a population’s decline (Korpimäki et al. 1991; Norrdahl and Korpimäki 1995, 2000). Bank voles are able to detect the odor of mustelids and change their behavior accordingly (Ylönen 1989; Jędrzejewska and Jędrzejewski 1990; Jędrzejewski and Jędrzejewska 1990; Mappes and Ylönen 1997; Mappes et al. 1998; Bolbroe et al. 2000; Pusenius and Ostfeld 2000; Ylönen et al. 2003). The least weasel, like other small mustelids, has a very potent anal gland secretion (Apfelbach et al. 2005) which has been shown to be interpreted by voles as a cue of predation risk (e.g., Ylönen et al. 2006; Haapakoski et al. 2012).

The study was conducted at the Konnevesi Research Station of the University of Jyväskylä, 70 km north of Jyväskylä. The study was conducted in the laboratory and the bank voles were trapped from the forests surrounding the research station (N 62° 41′ 18″, E 26° 17′ 12″) as well as in the forests near Oulainen (N 64° 17′ 56″, E 24° 49′ 35″). The voles were housed in solitary standard laboratory rodent boxes (43 × 26 × 15 cm^3^). Wood chips were used to keep the cages dry, hay was provided as bedding material, and rodent food pellets and fresh water were available ad libitum. Light: dark time ratio in the animal rooms was set to 18:6 h, which corresponds roughly to the natural light-dark regime during the experimental period. Four days prior to the experiment, the animals were removed from ad libitum food and put on a diet of poor-quality food, 3 g of millet per day, while meeting the basic energy needs of these animals (cf. Eccard and Ylönen 2006). The change to a poor diet was given as an incentive for the animals to keep foraging in the novel environment of our study systems and counteract neophobia expected in satiated animals (Amézquita et al. 2013; Näslund and Johnsson 2016). All applicable international, national, and/or institutional guidelines for the use of animals were followed and were approved by the animal experimentation committee of the University of Jyväskylä, permit number: ESAVI/6370/04.10.07/2014.

### Study system design

Six interview chamber systems were constructed in concordance with Bleicher (2014) and Bleicher and Dickman (2016) and Bleicher et al. 2018. Each system was constructed from a 30 cm diameter bucket (as a nest box) attached by 5 cm diameter, 30 cm long PVC tubing to three gray plastic storage bins (hereafter rooms) 40 × 30 × 23 cm high (Appendix S1). Each room was equipped with a square (19 × 19 × 10 cm high) box (henceforth patch) with two 5-cm-diameter holes drilled in the side to allow the vole access (Appendix S2). Each patch was filled with 1 l of sand and was set with 1.5 ± 0.02 g of millet. The total amount of food in the system equaled one and a half times the daily needed energy for a foraging vole (cf. Eccard and Ylönen 2006).

The rooms were equipped with a cue-box 11 × 11 × 6 cm attached to the roof of the room to create different treatments: weasel bedding (wood shavings, urine, hair, and fecal matter), rabbit (*Oryctolagus cuniculus*) bedding, and a control (cf. Sundell et al. 2008). Each cue-box was replenished with fresh bedding daily. The cardinal directions in which each treatment was placed was randomized between systems.

On direct predator exposure nights, two of the three-room interview systems (Nos. 5 and 6), were converted to four-room systems adding a room with hardware cloth screen directly adjacent to a cage (30 × 52 × 26 cm) in which a live weasel was caged. The weasel was caged only during experiments and was released into a larger 2-m-long holding pen when not in use (Appendix S3). To adjust for the larger systems, we decreased the amount of food per patch to 1.1 ± 0.02 g and to maintain the same encounter rate with a food item, decreased the amount of sand in the patch to 0.75 l. To avoid the sound of the caged weasel traveling throughout the system, the rooms adjacent to the weasel cage were located in a different lab-room with the PVC tubes drilled through the wooden wall between the lab-rooms.

We define an experimental round as the time an animal spent in a system on a given night. At the start of every round, a single vole was placed in the nest box and had access to each of the different patches via the PVC tubing. Each vole was allowed 2 h to forage, following the protocol of Bleicher (2012), and the expectation that this allowed sufficient time for voles to move between, and forage in, the different treatments while not giving enough time for habituation to the treatments. For the short period of time, diverging from the “normal” patch-use protocol (Bedoya-Perez et al. 2013) was aimed at getting the initial response of the animal, i.e., its “gut feeling” and not measure its ability to understand how we are manipulating it on the long run. We did not control for conspecific odors but avoided cross-contamination by consistently keeping each olfactory treatment in the same rooms consistently.

Brown (1988) stated that an animal foraging in a patch will quit harvesting when the costs associated with resource harvesting coupled with the costs associated with predation risk equal the energetic value of the patch as perceived by the forager. As the vole depletes a patch, the diminishing returns render other patches more valuable (a missed opportunity cost). The difference in missed opportunity costs drives animals across the landscape (between rooms) examining and comparing patches (Smith and Brown 1991; Berger-Tal and Kotler 2014). At the end of each round, the amount of resources the forager did not use in the patch, due to the aforementioned costs, is the giving-up density (GUD). We ran up to five rounds (of 2 h each) per “night” starting at 17:00 in accordance with activity patterns recorded by Ylönen (1988). The GUD as the measurement in our systems provides for data collection that is independent of the observer, a blinded design, thus allowing for avoidance of sampler bias.

Forty field-caught bank voles were interviewed (20 male and 20 females) for nine nights between the nights of May 6th–May 26th, 2018. Each individual vole was “interviewed” for nine nights in a row, randomizing systems and hours of the interview as to minimize the effects of time and location. Of the nine nights, the first two nights an animal was interviewed were without the live predator (hereafter pre-exposure). On the third and fourth nights, an animal was exposed to both a live weasel in addition to the olfactory cues (hereafter exposure). On the remaining five nights, the animal was exposed again to olfactory cues only (hereafter post-exposure). At the end of each round, the animal was removed and returned to its holding container and fed with extra 3 g of millet. Each of the patches was sieved and the weight of remaining resources recorded to obtain the GUD. The systems were reset after each 2-h round with fresh new patches and the next round run with a new vole.

#### Data analyses

For the analysis of the data in this paper, we did not use the GUD as in a traditional approach. To be able to compare the three-room and four-room system data we preferred to use the proportion of resources harvested (initial density-GUD/initial density). Because the runs are limited in time (2 h), the GUD does not reflect a quitting density but more of an initial response (if prolonged, we expected habituation and thus loss of relevance). Therefore, the traditional approach to the analysis of GUD data (e.g., St Juliana et al. 2011; Shrader et al. 2012), a general linear model (GLM), was not meaningful in this instance and would result in low explanatory power (we provide this weak analysis in Appendix S4). It is important to note that we excluded the live weasel treatment from all analyses (except for random forests analysis) to allow for a fully crossed experimental design. We felt confident in our ability to do this as only two voles had foraged in the live weasel patches, and those under 0.02 g of millet within the margin of error. For the same reason, we also only used the first two nights of the post-exposure rounds (marginal variation in 5 days post-exposure Appendix S4).

As an alternative, we used a combination of three statistical approaches. First, we ran log-linear tabulations for foraging activity (foraged vs. unforaged patches) (cf. Bleicher et al. 2016). We tabulated the data as a factor of chronology (pre-exposure, exposure, post-exposure) and treatment. We ran the analysis as a three-way contingency table using Vassar Stats calculator. We used this analysis to test the effects of each variable, the interaction of the two, and each nested within the other.

Second, we used Statistica© to run a series of tests of concordance as a repeated measure testing the response of individuals using the proportion of resources harvested as the dependent variable. Using Friedman’s tests of concordance, we first tested whether the vole’s foraging tenacity repeated the same pattern based on the chronology of the experiment. In the second test, we compared their response to olfactory cues. We also compared the foraging tenacity of individuals as factors of the interaction of treatment and chronological order, as well as treatment nested within chronological order.

Last, we chose to run our data through a random-forest regression analysis. This Bayesian machine-learning test uses the data and repeated sampling to determine the importance of factors in the decision-making process of voles. In this analysis, we used a normalized proportion of resources harvested (within patch) using an arcsine × √ transformation as the dependent variable. We used chronology, treatment (including live weasel), sex, and round (night within chronology). This analysis generates a decision tree ranked by importance from the top node to the lowest importance in final nodes. The final nodes on the tree provide likely hypotheses for significant differences but do not constitute statistical pairwise comparisons.

#### Data availability

All data generated or analyzed during this study are included in this published article and its supplementary information files.

## Results

When we measured the individual’s response to risk using foraging tenacity and tested whether they were in agreement about the value of patches in the different treatments, we found that there was no concordance between the voles in response to the cues as a main effect (Table 1). However, we found concordance in the change in foraging tenacity between the three segments of the experiment (pre-, post-, during-exposure). Here, we found that the interviews during-exposure resulted in a mean rank, the lower the rank the greater the risk, of 1.31. This compared to 2.46 and 2.23 for pre- and post-exposure interviews, respectively. The interaction of treatment and chronology, ranking the nine subcategories, found concordance between the individuals as well.

**Table 1:** Compilation of Freidman’s tests of concordance

Variable	Nested factors	*N*	df	*X*_f_	*P*	*W*
Chronology		39	2	29.077	< 0.001	0.373
Treatment		39	2	3.273	0.195	0.042
Chronology (treatment)	Pre-exposure	39	2	1.66	0.436	0.021
	During-exposure	39	2	6.764	0.034	0.087
	Post-exposure	39	2	10.839	0.004	0.08
Chronology × treatment		39	8	54.645	< 0.001	0.175

*N* sample size, *df* degrees of freedom, *X*_*f*_ Friedman’s chi-squared, *P* probability value, *W* Kendall’s coefficient of concordance

When we measured concordance between treatments nested under each of the chronological interviews, we found concordance in risk assessment only during- and post-exposure. During the exposure, the voles ranked the weasel-cue treatment as the greatest risk with the rabbit and controls having similar ranks. Post-exposure, the voles ranked the control as the least dangerous and avoided both rabbit and weasel cues similarly.

**Fig. 1:**
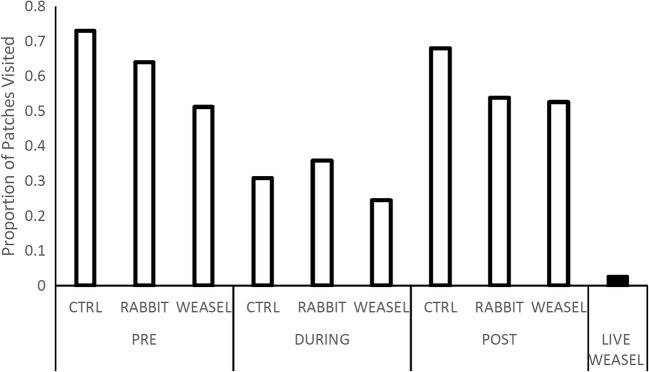
Cumulative proportion of patches harvested by voles in the systems. Each bar represents the foraging activity of 39 voles foraging for two consecutive nights (one female removed showing signs of pregnancy). On the *x*-axis, we state the olfactory treatments as collected at the different chronological states of the experiment, pre-, post-, and during-exposure to the live weasel. The value for the live weasel was excluded from the statistical analysis of activity patterns but is presented here for the comparative power it provides

**Fig. 2:**
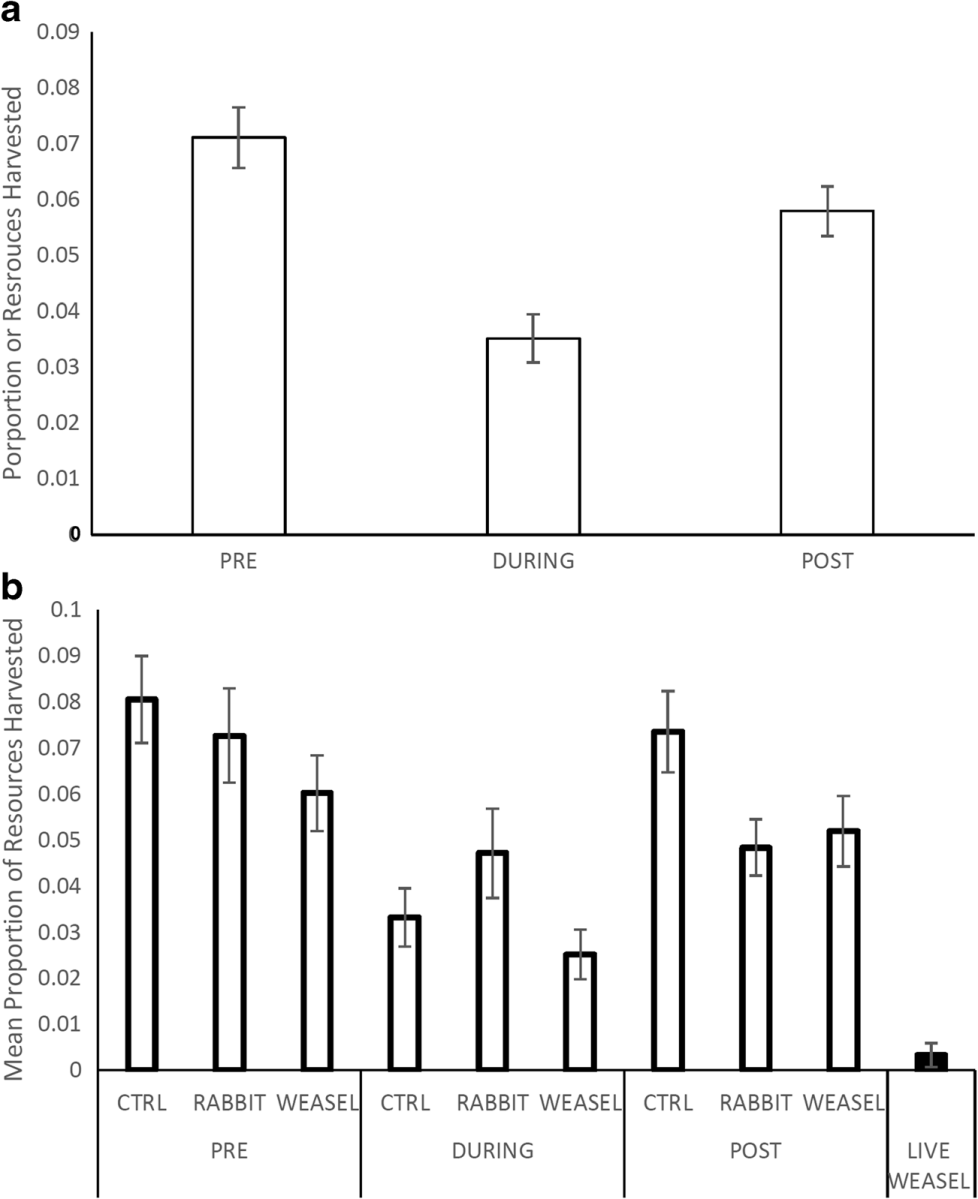
Mean proportion of resources harvested ± SE based on (A) the chronological order of interviews (*x*-axis) and (B) olfactory cue treatments nested under each chronological order. The value for the live weasel was excluded from the statistical analysis of activity patterns but is presented here for the comparative power it provides. Note that these values are the pre-normalized values which were transformed using a arcsine × sqrt transformation

Both the voles’ foraging activity (number of patches visited) and tenacity (proportion of resources harvested) were impacted similarly by the absence of the predator pre- and post-exposure (Fig.1 and Fig. 2A, respectively). The vole’s activity was lower in all types of patches when the weasel was nearby (Table 2). In addition, they foraged less in patches than on nights when the predator was absent (Appendix S4).

**Table 2:** Log-linear analysis using a three-way contingency table comparing the cumulative ratio of foraged to unforaged patches

Variable	*G* ^2^	df	*P*
Treatment	10	2	0.0067
Chronology	58.86	2	< 0.0001
Treatment × chronology	74.12	12	< 0.0001
Treatment (chronology)	15.28	6	0.018
Chronology (treatment)	64.14	6	< 0.0001

*df* degrees of freedom, *treatment* olfactory cue type, *chronology* order of interviews (pre-, during-, post-exposure)

In the three-way contingency table, the voles were more active in control patches as expected pre- and post-exposure. In the sham control (rabbit bedding), they were most active during nights of direct exposure to the weasel (Fig. 1). Proportionally, there was no difference in activity for weasel-cue treatment patches pre- and post-exposure. The major difference between the pre-exposure interviews to the post-exposure ones was the activity rate in the sham treatments decreasing from 64 to 54%.

Addressing the foraging tenacity, the proportion of food foraged in the patches was better addressed using a random-forest analysis (Table 3) than the traditional method of GLM (Appendix S4). The decision-tree risk estimates were low, 0.0217 ± 0.001 standard error and 0.025 ± 0.002 for the training and test of the model, respectively. This analysis ranked the variables by importance, i.e., the proportion of the decisions it influenced in the model. Chronology was the most important influencing 100% of decisions. Treatment influenced 85% of decisions while the sex of the vole influenced 17% and the repetition (round 1 or 2) influenced 16% of decisions.

**Table 3:** Tree structure for random-forest decision tree

No.	Child node 1	Child node 2	*N*	*μ*	Node var	Split variable	Split cons.	Split cat.1	Split cat.2
1	2	3	359	0.157	0.025	CHRONO		DURING	
2	4	5	183	0.102	0.018	TREATMENT		LIVE WEASEL	
3	6	7	37	0.005	0.001	ROUND	1.5		
4			16	0	0				
5			21	0.008	0.001				
6	8	9	146	0.127	0.020	SEX		MALES	
7	10	11	66	0.101	0.015	ROUND	1.5		
8			26	0.127	0.019				
9			40	0.084	0.011				
10	12	13	80	0.149	0.023	ROUND	1.5		
11			40	0.144	0.026				
12			40	0.153	0.019				
13	14	15	176	0.213	0.026	TREATMENT		WEASEL	RABBIT
14	16	17	128	0.194	0.025	SEX		MALES	
15	18	19	62	0.167	0.018	TREATMENT		WEASEL	
16	20	21	35	0.158	0.019	CHRONO		PRE	
17			15	0.111	0.019				
18			20	0.194	0.016				
19			27	0.178	0.016				
20	22	23	66	0.219	0.030	ROUND	1.5		
21	24	25	41	0.239	0.031	TREATMENT		RABBIT	
22			20	0.237	0.023				
23			21	0.241	0.039				
24			25	0.187	0.027				
25	26	27	48	0.263	0.027	CHRONO		POST	
26			17	0.194	0.029				
27			31	0.301	0.022				

*N* node sample size, *μ* mean normalized proportion harvested, *var* node variance, *cons*. constant, *cat*. category, *CHRONO* order of interviews (pre-, during-, post-exposure), *TREATMENT* cue type (weasel, rabbit olfactory cues, control, or live weasel)

**Fig. 3:**
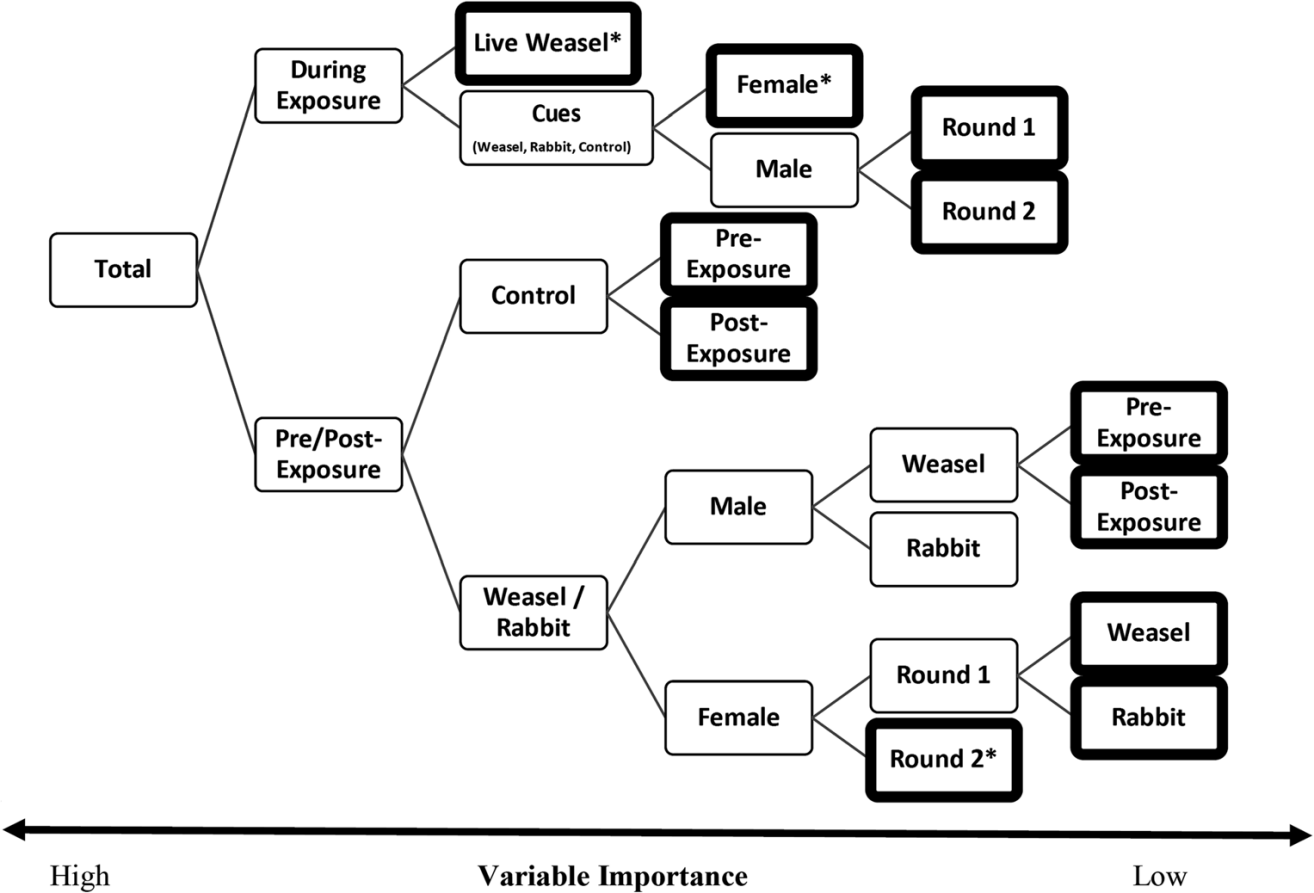
Decision-tree visualization based on a random-forest analysis using proportion of patches harvested (normalized using an arcsine × sqrt transformation). The further up the tree a split occurs the more important that variable is to the decision-making process of the voles. The asterisk denotes a terminal node (bold frame) which in the original tree produced further nodes; however, the difference in foraging was lower than 1% and was thus removed from the figure for clarity purposes

Reading the decision tree is from left to right (Fig. 3). The higher (further left) the split in the tree, the more important that split variable is in influencing the decision-making process of the foraging animal. The major split was chronology based, separating the exposure from the interviews where there were cues alone (Fig. 2B). We will present the results first for the direct exposure interviews and then in a separate paragraph address the pre- and post-exposure interviews.

Under direct exposure, only twice were the patches in direct view of the weasel foraged. Thus, the fact that this category resulted in a terminal node this early is suggestive of a high importance to that treatment. The analysis then revealed that there is no difference in response to all the other treatments. Females were as weary in the first and second night of an interview, foraging 14.4% and 15.3% of patches respectively. Meanwhile, males decreased foraging from 12% on the first night to 8% on the second.

Pre- and post-exposure, voles gave greater attention to the cue treatments. The model analyzes the dataset remaining after each split in the tree and chooses the next split based on the categories and variable that have the greatest variance in means. Therefore, we present both the important splits revealed in the decision-making of a population of voles (Table 3, Fig. 3), but also present the mean proportion of resources harvested within each split category. Pre-exposure, the control patches were foraged to a mean of 19.4%, but after exposure, the foraging increased to 30.1% (Figs. 2, and 3). The sexes then diverged on the response between the weasel and rabbit (sham) cues. Males foraged less, a mean of 16.7%, while females foraged resources to a mean 21.9%. For the females, the no significant difference appears between weasel (lower with 23.7%) and rabbit (higher with 24.1%) treatments on nights when no live predator is present. Males were more attuned to the differences between the rabbit and the weasel cues, foraging 17.8% and 15.8% of resources respectively. Post-exposure, the males took more risk with the weasel cues foraging 19.4% of resources compared with 11.1% pre-exposure.

## Discussion

The voles clearly made decisions regarding the use of space when interacting with olfactory cues. The information a forager gains from olfactory cues varies based on a number of environmental and temporal variables (Sih 1992; Lima and Bednekoff 1999; Gonzalo et al. 2009). The interpretation of the cues clearly impact the strategic behaviors of the foragers and the way the animals will balance the tradeoffs of food and safety (Brown et al. 1999; Bytheway et al. 2013; Bleicher 2017). This experiment provides a clear example of how the same cues vary in the information they provide over a short period of time.

We believe the strongest evidence supporting the fact that a cue changes its meaning is the lack of a main effect to the cue treatments in all three analyses. This suggests immediately that each cue is not interpreted in the same way at each of the chronological intervals. This is a reflection of the changes in the state the animal is in. The animals in this experiment were wild caught; however, they came into the experiment after a few weeks in a lab setting with ad libitum resources available to them.

At the first encounter with our systems, the animal arrives with a certain naiveté towards the cues. This could likely suggest that the animals may not have perceived a risk cue as relevant to their existence as lab animals. Previous laboratory studies show anti-predatory behavioral responses to olfactory cues manifested on larger scales in both movement and foraging decisions (e.g., Ylönen et al. 2006; Haapakoski et al. 2015). However, recent observations suggest that individuals approach and inspect any odor cue scented trap or box, inspecting its riskiness more carefully and adjusting responses. That study shows evidence for variation in personality traits within a population, and sex biases in risk taking (Korpela et al. 2011). Accordingly, the trend of decrease in foraging between the control and the weasel (with sham in between) suggests the weasel cue may be interpreted as suspicious, and the animals cautiously investigated those patches. The fact that the changes between treatments intensified in the repeated measures suggests that with the realization that the predator is a threat, all the other cues also change in value (Parsons et al. 2018).

This experiment provides other examples of the shifting value of information. The relative partiality of voles to forage in the rabbit-cue (sham) treatment during-exposure provides another strong example. The simplest explanation of this result lies in the fact that a prey leaves cues for its predators to interpret (Ylönen et al. 2003). On exposure nights, the voles are aware of the imminent danger lurking in the system. Therefore, the optimal strategy they can apply is to forage in the environment that masks their presence mixed with competitor cues. Similar to the idea of safety in numbers (e.g., Rosenzweig et al. 1997), a forager can hide behind the smell of a prey of higher caloric value. The rationale behind this strategy is that the predator would approach that environment in search of a different type of prey providing enough time for the less valuable forager to escape. A number of model papers by Lima and Dill (1990) and Brown (1992, 1999) suggest that evolution would drive prey to sacrifice resources in the form of competition due to risk of predation. This is even strengthened by empirical evidence of some species even cloaking their own odor as in the example of ground squirrel masking their odor towards snakes with the musk of other snakes (Clucas et al. 2008).

Post-exposure, the voles’ foraging and visitation in both weasel and rabbit-cue treatments were low while increasing in the control. Two possible explanations, separate or combined, would result in this observed decision. The first possibility is that the reduced foraging over the exposure nights resulted in animals with higher energetic needs, i.e., starved individuals. Hungry individuals can be forced to move large distances to find resources (e.g., Haythornthwaite and Dickman 2006); however, they are also likely to exploit resources to a greater extent when they encounter a valuable patch (Raveh et al. 2011). The opposite explanation would suggest that after experiencing the existential fear during the exposure nights, the voles are now more sensitive to the cue of the predator and fear being flushed out by the “competition” (the non-present rabbit) into a risky situation. While this second possible hypothesis stands in contradiction to the explanation we gave for the observation we made on exposure nights, the amount foraged on those nights was significantly lower. We will not be able to tease the strategic reasoning the voles are taking in this instance, and this could be used for future experimentation managing the animal’s energetic state.

In addition to the overwhelming effect of the live predator, the caged weasel, the random-forest analysis suggested sex-dependent differences. Similarly, to energy state-dependent decision-making, sexual selection drives individuals of the opposite sexes to make decisions related to mate finding and territoriality. In the bank vole, females are territorial (Koskela et al. 1997) while males move large distances in search of mates (Kozakiewicz et al. 2007). As a virtue of living a sessile life, as females do, the risk associated with cues are more relevant. Therefore, we do not see habituation from one night to the next in the initial interview. However, when the cues persist—and the level of risk is tuned up by exposure the predator—the opposite is correct. Post-exposure, we observe that females decreased foraging on the second night of the interview.

The transient nature of males means they are encountering the cues, and are assessing them, as novel cues each time. We therefore find no habituation to the treatments in the systems, but instead an increase in perceived risk from the first to the second night of each interview. In direct contact with the weasel, the entire harvest rate of males drops by 25% from the first to the second night of the interview. To the males, moving on larger scales, the repeated encounter with the weasel may be a measure of greater weasel encounter chances. Previous studies show that males respond to mammalian predators by decreasing their exploratory behavior (Norrdahl and Korpimäki 1998).

Our setup allowed us to examine how the interpretation of a cue changed based on the change of individual voles’ environment and state from partial naiveté to predators through a period of heightened risk to gradually fading memory of danger in a post-predator environment. We found that by examining the relationship between cues simultaneously, we can begin to extract some of the building blocks of the strategic behaviors exhibited by a forager. While not surprising that the responses to cues varied, the novelty here is that it calls the interpretation of predation cues into scrutiny. The findings here particularly question the way we administer predation stress using olfactory cues. We are approaching the time where we will be able to observe the responses an individual makes as it stimulates neurobiological circuits. We must reckon with the fact that an individual’s cognition and neural processes which takes places in animals’ brain affect the interpretation of cues to a much greater extent than we acknowledge in our simplified models. We, as a scientific community, need to consider “fear” responses in future experimentation.

Further, this framework allows us to ponder the value of the information prey gather from encountering novel predators in the invasive species scenarios we started with. While a major emphasis is put in studies to why prey are naïve to the cues left by a predator, we suggest that some effort be directed towards the investigation of what the animals are actually interpreting these cues to mean. Finally, due to the inherent dependency of the olfactory cues on the predators that leave them behind—the movement of the predator generates a temporal and spatial pattern in which the prey navigate, forage, and mate. We show here the result of the way prey respond in an evolutionary game, where the prey evolved the optimal ability to interpret the innuendos of the odor left behind by their predator in a complex environment.

## Electronic supplementary material

ESM 1 (XLSX 79 kb)

ESM 2 (DOCX 2073 kb)
