# Supplementary material for: Olfactory cues and the value of information: voles interpret cues based on recent predator encounters
Source: Behav Ecol Sociobiol. 2018 Nov 26;72(12):187. doi: 10.1007/s00265-018-2600-9 (PMC6267667; doi:10.1007/s00265-018-2600-9)
Supplement: Supplementary file 2 — (DOCX 2073 kb) [file 265_2018_2600_MOESM2_ESM.docx]

ESM 1 – Photographs of the interview systems as set up at the Konnevesi Research Station, note that systems 5 & 6 have a fourth prong extending to another lab-room to the location of the caged weasel. The boxes on top show the dimension of the foraging patches used in the experiment.


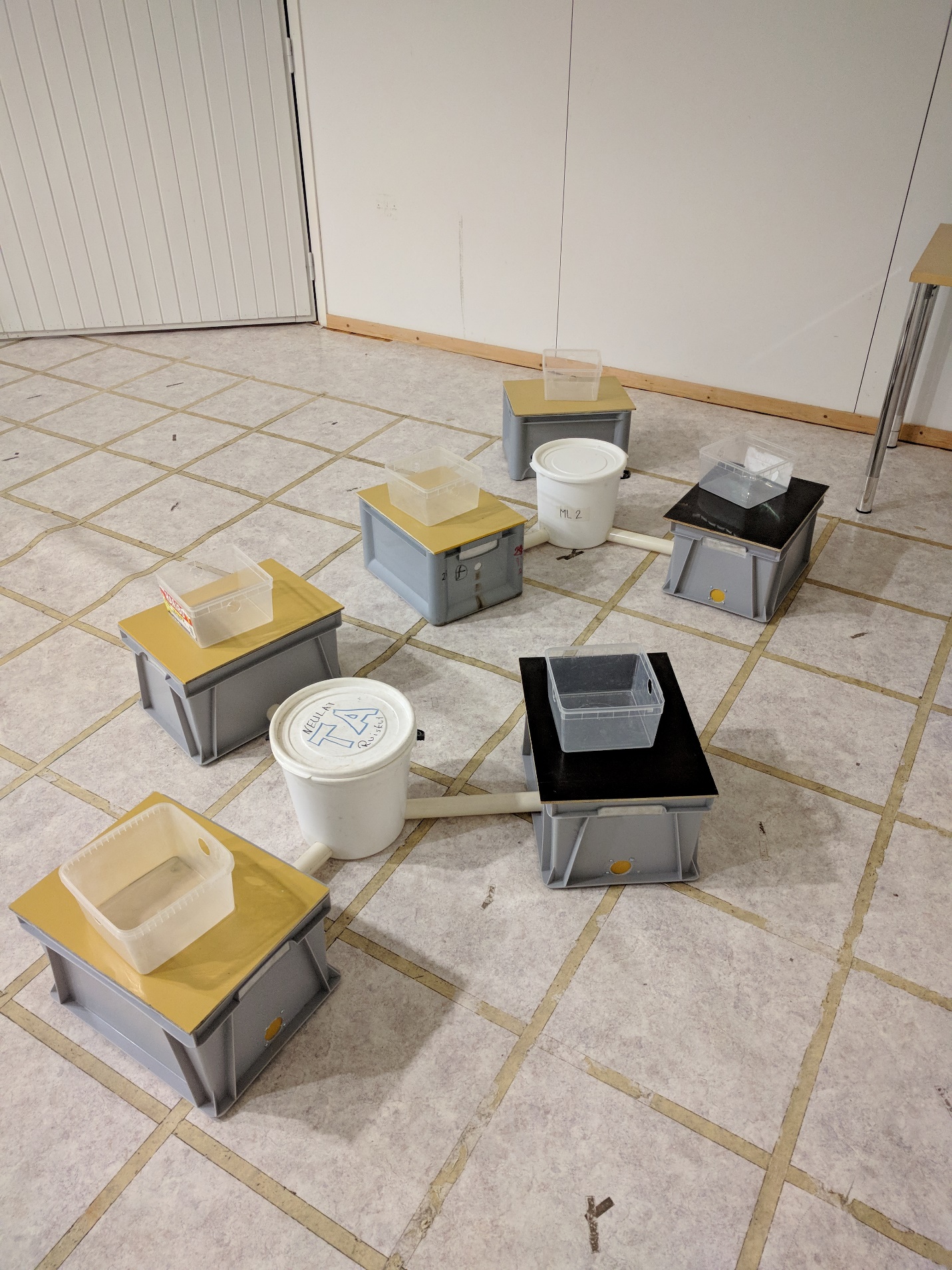


**ESM 2. System Design**

A. Foraging patch set-up as used for this experiment. Each box contained 1 liter of sand and 1.5 grams of millet mixed in with the sand. For the exposure nights we kept the same ratio, but reduced both sand and millet amounts to 0.75 l and 1.1g respectively to accommodate for the added room while not adding energy for the voles in the new setup. B. A vole at the end of an experimental round.


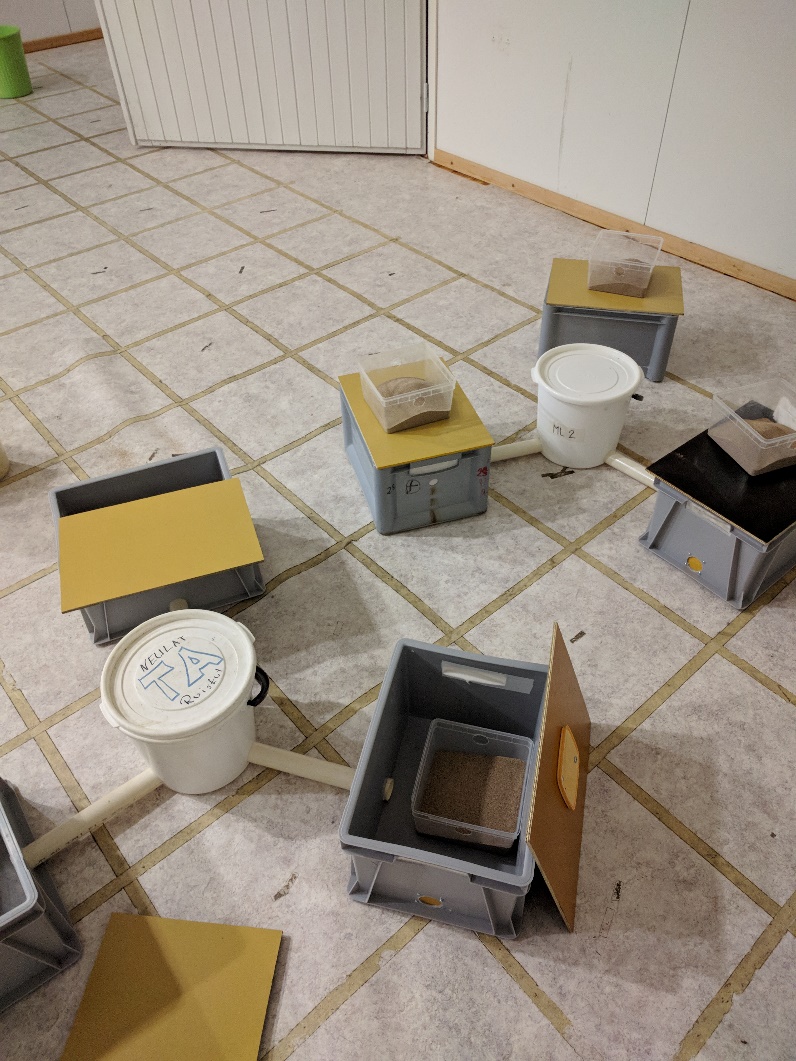
**
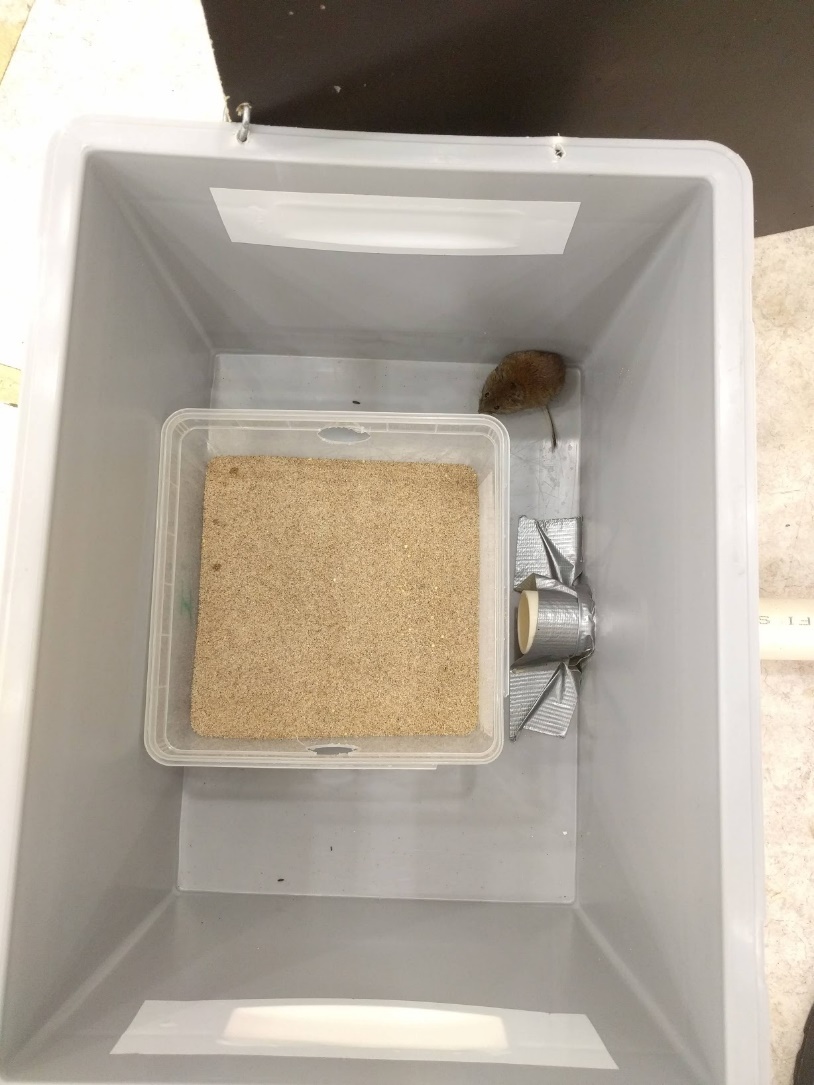
**

ESM3. Photographs of the A. Weasel Cage Set Up with the latch closing the exposure part for experiments. B. the weasel as seen from a vole’s perspective as it enters the exposure treatment.


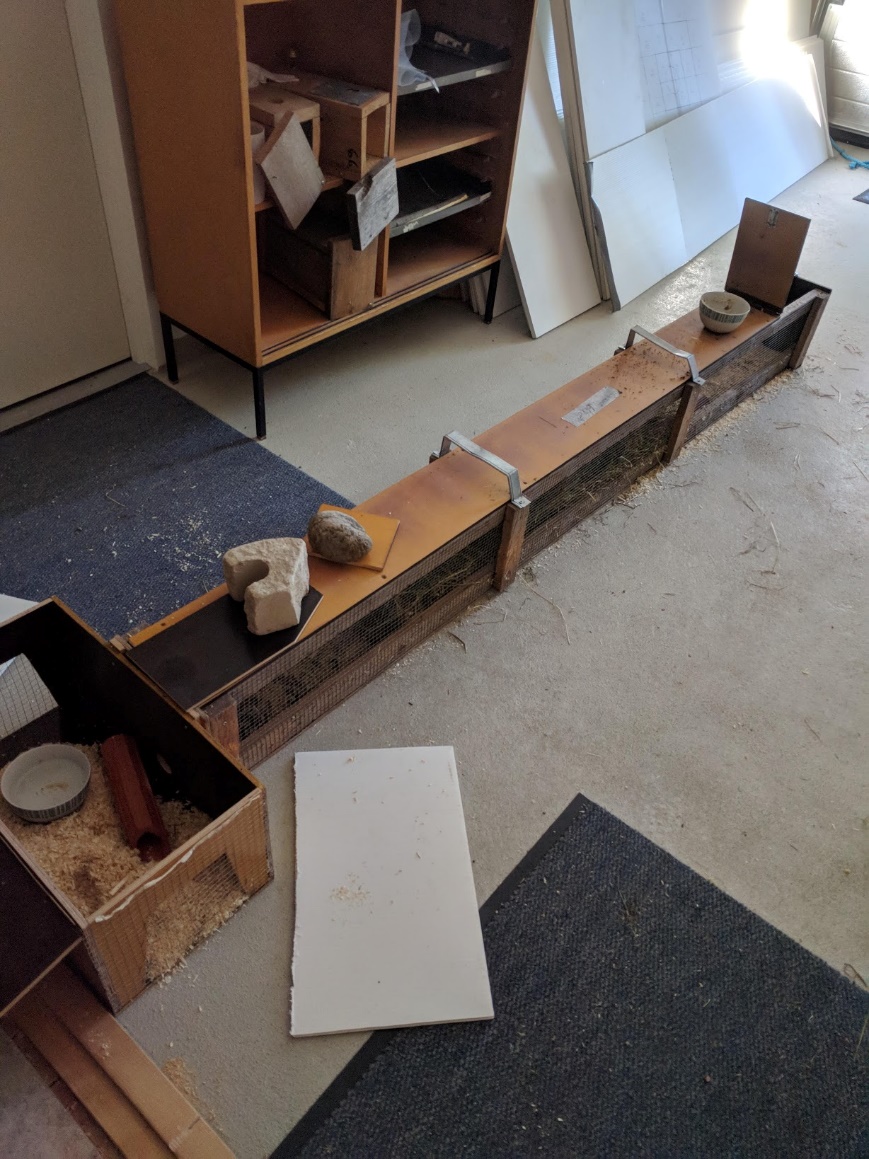

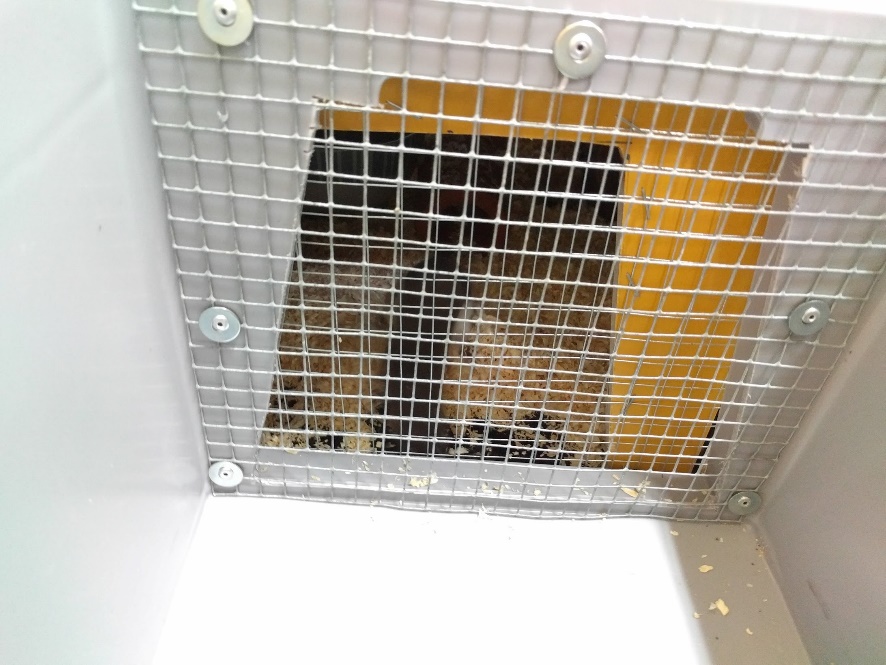


**ESM 4A.** **Excluded Statistical Analysis.**

General Linear Model Analysis results excluded from paper. The model using averaged the proportion harvested per system, per round of the night (up to 5), per night. This averaging was preformed to remove vole individuality. The means were then transformed using an arcsine × sqrt transformation to normalize the data prior to analysis. The final model’s N=132 and the Multiple-R^2^= 0.306.

| **Analysis of Variance** | | | | | |
| --- | --- | --- | --- | --- | --- |
| **Source** | **Type III SS** | **Df** | **Mean Squares** | **F-Ratio** | **p-Value** |
| CHRONOLOGY | 0.283 | 2 | 0.141 | 14.586 | 0.000 |
| TREATMENT | 0.043 | 2 | 0.022 | 2.232 | 0.112 |
| CHRONOLOGOY × TREATMENT | 0.056 | 4 | 0.014 | 1.442 | 0.224 |
| TIME | 0.133 | 1 | 0.133 | 13.681 | 0.000 |
| ROUND | 0.015 | 1 | 0.015 | 1.561 | 0.214 |
| Error | 1.173 | 121 | 0.010 |  |  |

Abbreviations: SS (sum of squares), df (degrees of freedom), Chronology (order of interviews pre-, during-, post-exposure), Treatment (Olfactory Cue Source), Time (start time of the round), Round (the nigh of the interview within the chronological order (first or second night).

**ESM 4B. Excluded Statistical Analysis.**

Variations represented by box plots by the start time of each round of the experiment with the normalized (arcsine × sqrt transformed) proportion harvested as a dependent.
